# Macro- and Microvascular Function in Middle-Aged Individuals with Low Cardiovascular Disease Risk

**DOI:** 10.3390/jcm11236962

**Published:** 2022-11-25

**Authors:** Sunni Patel, Hala Shokr, Adam Greenstein, Doina Gherghel

**Affiliations:** 1 Vascular Research Laboratory, Ophthalmic Research Group, College Health and Life Sciences, Aston University, Birmingham B4 7ET, UK; 2 Pharmacy Division, Faculty of Biology, Medicine and Health, The University of Manchester, Manchester M13 9PL, UK; 3 Division of Cardiovascular Sciences, The University of Manchester, Manchester M13 9PL, UK

**Keywords:** microvascular function, retina, vascular endothelial, flow-mediated dilation

## Abstract

Aims: To investigate the microvascular function in apparently healthy individuals showing signs of early macrovascular endothelial dysfunction. Methods: Healthy participants aged between 30–55 years were recruited for the present study. Baseline measurements included body-mass index (BMI), waist-to-hip ratio (WHR), 24-h blood pressure, as well as fasted venous glucose, triglycerides (TG) and cholesterol (HDL, LDL and total). Brachial artery reactivity was measured using the flow-mediated dilation (FMD) technique and retinal vessel reactivity was assessed by using the Dynamic Retinal Vessel Analyser (DVA) in all individuals. The enrolled participants were separated in two groups, based on either a reduced (group 1: <5%—*n* = 53) or a normal FMD response (group 2: 7–10%—*n* = 47). Results: Individuals exhibiting reduced FMD responses showed a reduced baseline-corrected microvascular arterial dilation response to flickering light (*p* = 0.039). In addition, they also exhibited a reduced arteriolar maximum dilation (*p* = 0.034), as well as a longer dilation reaction time (*p* = 0.048) and a lower dilation amplitude (*p* = 0.042) when compared to those with normal FMD values. Conclusion: In otherwise healthy middle-aged individuals, early signs of vascular dysfunction are reflected simultaneously at both macro- and microvascular levels.

## 1. Introduction

Healthy vascular endothelial cells (EC) support the cardiovascular function by promoting adaptive vasodilation, inhibiting platelet aggregation, white blood cell adhesion, and smooth muscle cell proliferation [1]. Therefore, whenever it occurs, vascular endothelial dysfunction (VED) will result in development and progression of atherosclerosis, tissue ischaemia and vascular thrombosis, all factors that lead to various circulatory pathologies [2,3].

It has been shown that EC are different in different types of vascular beds [4]. In addition, there is a heterogeneity in the endothelial function not only in different vascular beds, but also in various segments of the same vessel [5]. Despite this, VED is a parameter important to assess not only as an early marker for cardiovascular disease (CVD) risk but also as a marker for the efficacy of various vasoactive medication.

Systemic vascular function is usually quantified by assessing the endothelium-dependent relaxation of the brachial artery after increase in shear stress, as determined using the flow-mediated dilation (FMD) technique [6,7]. An abnormal FMD represents a well validated marker for increased CVD risk [6,8]. Nevertheless, it has become apparent that structural and functional changes in other vascular beds, especially at the microcirculatory level, could provide even earlier and better predictions for the development of vascular disease [9,10,11,12,13,14,15,16]. Therefore, beside advancements in understanding the physiology of microvascular function, many efforts are out into to the development of novel methods and refinement of old techniques for the assessment of these type of vessels [11]. Among these, the quantification of retinal vascular dilation and constriction has already been identified as a good preclinical marker for various systemic vascular disorders [12,15,16,17] even in apparently healthy individuals [12,18,19].

It has previously been proposed that macro- and microvascular beds should be imaged in parallel and as early as possible, given their capacity to provide important, complementary information [11]. Therefore, the aim of the present study was to investigate possible signs of microvascular function abnormalities in apparently healthy individuals showing signs of early VED as identified by the gold standard technique, FMD.

## 2. Materials and Methods

The study cohort consisted of healthy normoglycemic, and normotensive participants aged 30–55 years that were screened and recruited from the Health Clinics at Aston University, Birmingham, UK.

Exclusion criteria were family history of DM, a positive diagnosis of cardio- or cerebro-vascular disease, (coronary artery disease, heart failure, arrhythmia, stroke, transient ischaemic attacks), peripheral vascular disease, severe dyslipidaemia (defined as plasma TG > 6.00 mmol/L or cholesterol levels > 7.00 mmol/L), diabetes, as well as other metabolic disorders or chronic diseases that required treatment [9,19,20]

Furthermore, subjects were excluded if they had a refractive error of more than ±3 Dioptre Spherical and more than ±1 Dioptre Cylindrical equivalent, intraocular pressure (IOP) >24 mmHg, cataract, or any other media opacities, as well as if they had a history of intraocular surgery or any form of retinal or neuro-ophthalmic disease affecting the ocular vascular system [12,18].

Written informed consent was obtained from all participants and ethical approval was sought from local academic and NHS ethical committees. The study was designed and conducted in accordance with the tenets of the Declaration of Helsinki.

According to an already established procedure when examining endothelial function, female participants were asked to fill in a validated menstrual cycle questionnaire and their investigations were carried out during the first week of the menstrual cycle (follicular phase).

### 2.1. Blood Analyses

All participants were asked to fast and refrain from caffeine, alcohol, chocolate and carbonated drinks and to not exercise for 12 h prior to the date of the study. All blood samples were obtained by a qualified phlebotomist in the morning, between 9 a.m. and 10 a.m. Fasting plasma glucose, TG, total and HDL-C were measured using standard routine laboratory techniques using the Reflotron Desktop Analyser (Roche Diagnostics, Burges Hill, UK).

### 2.2. Ambulatory Blood Pressure Measurements

Systemic BP was measured using a 24-h computer-operated ambulatory BP monitor (Cardiotens-01, Meditech Ltd., Hungary) for each subject. Measurements were performed in ambulatory conditions and programmed to measure BP oscillometrically every 15 min during the subject’s active period and every 30 min in the passive period. Mean arterial pressure (MAP), as a means of describing cardiac output function in relation to arteriolar resistance was calculated according to: (⅔ × DBP) + (⅓ × SBP) [16].

### 2.3. CVD Risk Calculation

Framingham risk score (FRS) for CVD was also calculated using the National Heart, Lung and Blood Institute (NHLBI) worksheet, which is based on age, gender, CHOL, HDL-c, SBP, treatment for hypertension, smoking, and diabetes [21,22]. Only individuals typically stratified as low risk (<10%) were included in the present study [23].

### 2.4. Vascular Function Assessments

a. Macrovascular function

Brachial artery FMD was measured using high-resolution CDI ultrasonography, with a 7 mm 8MHz linear-array (Siemens; Acuson Sequoia, UK) and the diameter was continuously measured using wall-detection specialised artificial neural networking software (VIA^®^ Software, UK) from the anterior to the posterior interface between the media and adventitia on a personal computer [24]. The brachial artery was imaged above the antecubital fossa in the longitudinal plane and a segment with clear anterior and posterior intimal interfaces between the lumen and vessel wall was selected for continuous 2D greyscale imaging. According to a published protocol [25], a baseline rest image was acquired for two minutes, and thereafter, arterial occlusion created by cuff inflation to suprasystolic pressure (50 mmHg above systolic) for a standardised five minutes. The longitudinal image of the artery was recorded continuously for two minutes (min) after cuff deflation. Following 10 min of rest another image was acquired to reflect the re-established baseline conditions. An exogenous sublingual 0.3 mg glyceryl trinitrate tablet (GTN) was then given to determine the maximum obtainable vasodilator response, and to serve as a measure of endothelium-independent vasodilation reflecting vascular smooth muscle function [25]. FMD was then expressed as a percentage of the maximal artery dilation (MD) during hyperaemia from the baseline absolute diameter (AD). Furthermore, the brachial artery diameter fluctuations (BDF), GTN-induced dilatory response relative to re-established diameter (GID) and the ratio between FMD and GID (FMD/GID) calculated to determine differences in vascular endothelial or smooth muscle cell response.

b. Microvascular function

Retinal vessel reactivity was measured using the DVA (Imedos; GmbH, Jena, Germany). All measurements were performed in one unselected eye for each subject, between 8:00 and 11:00 a.m. in a quiet, temperature-controlled room (22 °C). Following full pupil dilation with Tropicamide 1% (Minims; Chauvin Pharmaceuticals Ltd., UK) a region of interest encompassing vessel segments of approximately 500 µm was chosen.

Retinal diameters were assessed continuously over 350 s according to an accepted and widely used protocol [26]. In short, the whole measurement duration encompasses 350 s that consists of 50 s baseline followed by three 20-s cycles of flicker and 80 s of recovery.

**Figure 1:**
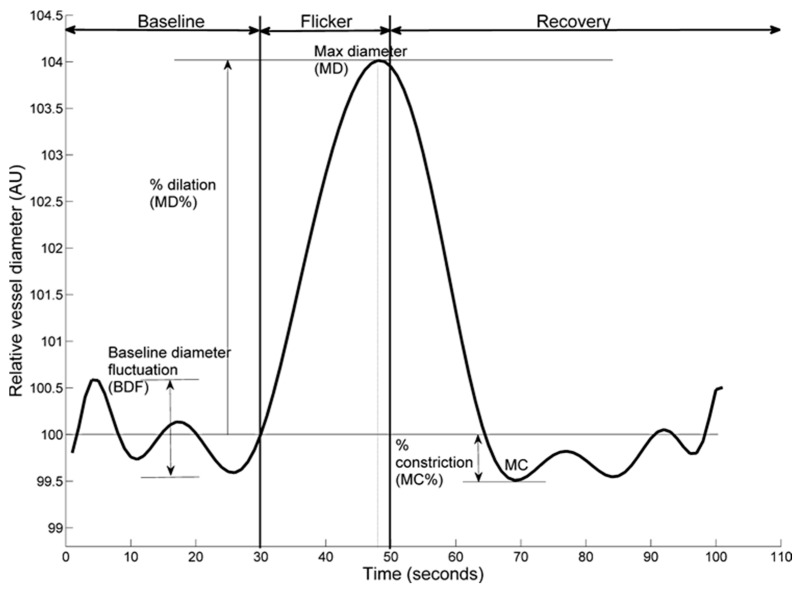
Example dynamic retinal arterial vessel response profile displaying parameters calculated and used in analysis. Percentage change in diameter from baseline to maximum (MD%) calculated as percentage increase in vessel diameter from baseline to maximum. Percentage constriction below baseline (MC%) calculated as percentage constriction below baseline following the point of maximum dilation.

The following retinal vessel reactivity and time course parameters, collectively known as Sequential and Diameter Response Analysis (SDRA), were then calculated (Figure 1). The differences between maximum and minimum baseline vessel diameter was termed as baseline-diameter fluctuation (BDF), the maximum diameter (MD) was used to describe the maximal vessel dilation in response to flicker light stimulation expressed as a percentage from baseline, the time taken (seconds) to reach the maximum vessel diameter during twenty second flicker exposure was termed as MD reaction time (MDRT), the minimal vessel diameter within thirty seconds of the recovery period was calculated as a percentage to baseline and expressed as the maximum constriction (MC) whilst the time taken (seconds) to reach maximal vessel constriction was termed maximum constriction reaction time (MCRT), and finally, the difference between maximal dilation and constriction responses was termed as the dilation amplitude (DA) [15,18].

### 2.5. Power Calculation and Statistical Analysis

Based on previous studies, normal expected retinal arterial responses to flicker-light stimulation have been around 6.9 ± 2.8% [27,28]. Additionally, vascular risk studies have reported FMD values of 7–10% in healthy controls [29,30]. As the study design was multi-factorial in nature it was calculated that *n* = 40 was sufficient to provide 90% power with an alpha of 0.05. Furthermore, the sensitivity and reproducibility of the techniques in healthy subjects has been reported previously [31,32].

All analyses were performed using Statistica^®^ software (version 13, StatSoft Inc., Tusla, OK, USA). Distributions of continuous variables were determined by the Shapiro-Wilks test. In cases where normality of the data could not be confirmed appropriate data transformations were made or non-parametric statistical alternatives were used. Univariate associations were determined using Pearson’s (normally distributed data) or Spearman’s method (non-normally distributed data), and forward stepwise regression analyses were performed to test the influence of clinical parameters and circulating markers on the measured vascular reactivity variables. Differences between groups were subsequently assessed using one-way ANOVA or ANCOVA, as appropriate, followed by Tukey’s post hoc analysis [16]. A *p* value of <0.05 was considered statistically significant, unless stricter criteria were adopted for within-group and multiple comparisons (*p* ≤ 0.01 to account for multiple comparisons and thereby minimise bias towards Type II errors).

## 3. Results

A total of 125 subjects were screened for eligibility. Due to poor data acquisition, 25 subjects were excluded. The remaining 100 subjects were then separated into 2 groups, using a to widely accepted selection, whereby a normal FMD response was defined as values between 7–10% and an attenuated response, as values ≤5% [31] (Table 1). There were no statistically significant differences between the number of men and women included in either group 1 or 2 (*p* > 0.05).

Baseline characteristics of both groups are presented in Table 2. There were no statistically significant differences in all anthropometric measures taken to determine the demographics between both groups (*p* > 0.05).

Retinal vascular reactivity values for each group of the study groups are presented in Table 3. There were no significant differences between the 2 study groups with regard to baseline arterial diameters (AD), baseline-diameter fluctuations (BDF), and constriction values post-flicker (MC and MCRT) (all *p* > 0.05). Equally, there were no significant differences found in venous responses between the 2 groups (*p* > 0.05).

**Figure 2:**
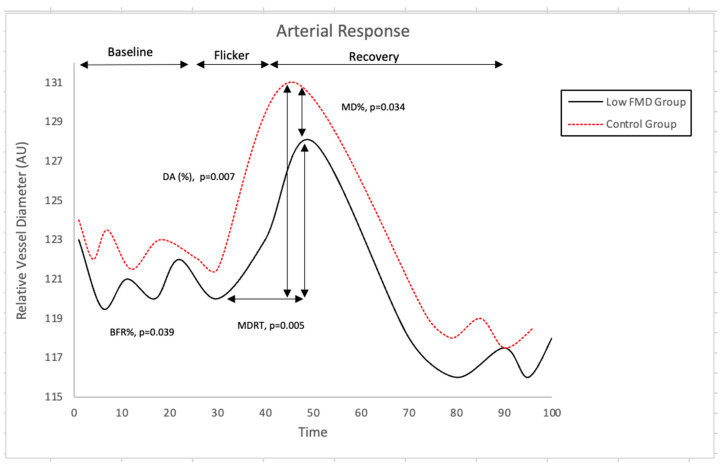
Comparison of Retinal Arterial Response Profile Across Groups. Abbreviations: AU, arbitrary units; BFR: baseline-corrected flicker response; DA: dilation amplitude; MD%: maximum dilation percentage; MDRT: reaction time to reach maximum diameter to flicker stimulation. There was, however, no correlation between the measured parameters at the macro- and microvascular level (all *p* > 0.05).

Nevertheless, individuals exhibiting reduced FMD responses showed a reduced baseline-corrected microvascular arterial dilation response to flickering light (*p* = 0.039). In addition, they also exhibited a reduced arteriolar maximum dilation (*p* = 0.034), as well as a longer, albeit borderline, dilation reaction time (*p* = 0.048) and a lower dilation amplitude (*p* = 0.042) when compared to those with normal FMD values (Figure 2).

## 4. Discussion

The present study reported, for the first time, that in apparently healthy individuals with low CVD risk according to established calculators, microvascular abnormalities are present in those that show signs of subclinical macrovascular dysfunction as assessed using the FMD. Nevertheless, there was no correlation between the vascular function response at the macro- and microvascular level in our participants.

Although macro- and microvascular circulatory beds are governed by different physiological mechanisms, a reduced basal NO availability can be the common denominator for the occurrence of endothelial dysfunction measurable at all levels [33,34]. Indeed, although retinal microvascular function mainly occurs due to an increased neural activity [35], flicker-induced vasodilatation may also be a measure of endothelial function [36], due a NO-related mechanism similar to that governing the FMD response [37]. Therefore, it can be expected that in case of widely spread endothelial dysfunction, the two type of measurements will show, at least in part, similar changes. Indeed, a previous study performed in healthy volunteers and in patients with various CVD risk found some, albeit weak, correlations between the retinal vascular function and FMD responses [35]. Despite the reported results, however, in terms of correlation between the functionality of the two vascular beds, our study failed to detect any relationship between the assessed FMD and RVA parameters. This can be due to the fact that we included only those individuals with a low CVD risk as determined using the FRS [18,23]. As such, whilst changes in the two vascular systems may go in parallel in individuals with low CVD risk, they will not exhibit, however, a direct relationship due to various physiological differences and influences that govern their functionality. Nevertheless, when circulatory pathologies will become more advanced, some degree of correlation between functional changes could occur at all levels. Indeed, the function of diseased vessels could possibly defy the local physiological rules and behave in a manner that is, somehow, similar across the entire body [12,18]. These complex observation and hypotheses need more investigations. Nevertheless, it is interesting that, for both vascular beds, the functional abnormalities were recorded in the amount of dilation post-provocation. Because brachial artery FMD is considered to represent an endothelium-depended dilation, which mainly depends on NO bioavailability [35], it can be hypothesised that a similar mechanism was responsible, at least in part, for the changes recorded at the retinal microvascular level. In addition, our findings could indicate that the assessment of retinal microvascular function is a suitable indicator of the systemic vascular endothelial function. Indeed, we have previously reported that the assessment of retinal microvascular function represents a method able to measure early vascular changes that possibly point out early endothelial dysfunction and future risk of cardiovascular pathologies in individuals with and without overt clinical symptoms [12,18,36]. The current findings also strengthen our previous reports. Moreover, by providing an integrated and dynamic analysis of vascular function that is, indeed, specific for each individual, retinal vessel reactivity could also be used for profiling a so-called individualized vascular risk for CVD [16,23,24,37]. This approach could be promising in furthering the concept of early vascular screening and prevention strategies, an important step towards the development of individualized and targeted endothelial therapies that can improve the lifetime risk in individuals that were traditionally considered low-risk [12,16,18,23].

## Figures and Tables

**Table 1:** Brachial Artery Reactivity Assessments.

	↓ FMD [*n* = 53]	Controls [*n* = 47]	*p*-Value
Brachial Artery			
AD (mm)	4.16 (3.39–4.65)	4.03 (3.41–4.64)	0.328
BDF (mm)	0.34 (0.17–0.42)	0.36 (0.21–0.44)	0.742
MD (mm)	4.32 (3.56–4.67)	4.53 (3.84–5.16)	0.200
FMD (%)	1.80 (0.39–4.27)	10.28 (7.25–11.60)	-
GTN			
GTN-MD (mm)	5.04 (4.14–5.62)	5.09 (4.62–5.60)	0.800
GID (%)	21.01 (14.19–28.34)	24.66 (16.58–31.86)	0.235

(↓ FMD—reduced ≤5% FMD group, Controls—FMD 7–10%). Values quoted in mean (IQR). Abbreviations: AD: absolute diameter; BDF: baseline diameter fluctuation; MD: maximum diameter response; FMD: flow-mediation dilation response; GID: GTN-induced dilation; FMD/GID: FMD/GID ratio.

**Table 2:** Baseline Characteristics of the Study Population.

	↓ FMD [*n* = 53]	Controls [*n* = 47]	*p*-Value
Demographic Data			
Age (years)	42.5 ± 11.6	40.1 ± 11.9	0.362
Weight (kg)	79.2 ± 16.0	76.0 ± 15.4	0.245
BMI (kg/m^2^)	27.5 ± 5.0	26.1 ± 4.5	0.084
WHR (AU)	0.95 ± 0.07	0.94 ± 0.12	0.413
SBP (mmHg)	122 ± 14	119 ± 14	0.321
DBP (mmHg)	76 ± 10	76 ± 10	0.673
MAP (mmHg)	92 ± 11	90 ± 11	0.486
IOP (mmHg)	13 ± 3	14 ± 3	0.077
Metabolic Data			
Glucose (mmol/L)	5.60 ± 0.47	5.51 ± 0.62	0.424
2-h GTT (mmol/L)	7.21 ± 2.11	7.08 ± 2.18	0.764
TG (mmol/L)	1.17 ± 0.47	1.39 ± 0.78	0.063
HDL Cholesterol (mmol/L)	1.20 ± 0.35	1.15 ± 0.39	0.417
LDL Cholesterol (mmol/L)	2.64 ± 0.99	2.63 ± 0.78	0.900
Total Cholesterol (mmol/L)	4.38 ± 0.93	4.41 ± 0.84	0.858

(↓ FMD—reduced ≤5% FMD group, Controls FMD 7–10%). Values quoted in mean ± SD. Abbreviations: SBP: systolic blood pressure; DBP: diastolic blood pressure; MAP: mean arterial pressure; IOP: Intra-ocular pressure; GTT: glucose tolerance test; TG: triglyceride; HDL: high-density lipoprotein; LDL: low density lipoprotein.

**Table 3:** Average Retinal Arterial and Venous Measures for Both Groups.

	↓ FMD [*n* = 53]	Controls [*n* = 47]	*p*-Value
ARTERY			
AD (AU)	122.28 (111.80–130.40)	124.00 (112.40–135.05)	0.581
BDF (AU)	4.88 (3.38–5.24)	5.60 (3.47–7.19)	0.157
MD (%)	4.46 (3.01–5.12)	5.49 (3.40–7.18)	0.034 *
MDRT (secs)	19.9 (15.8–23.1)	17.5 (12.0–21.3)	0.048 *
MC (%)	2.97 (1.34–3.66)	3.26 (2.04–4.47)	0.474
MCRT (secs)	20.1 (18.0–22.5)	20.3 (17.3–23.7)	0.821
DA (%)	7.43 (5.62–8.69)	8.74 (5.90–10.96)	0.042 *
bFR (%)	1.87 (0.35–3.69)	3.21 (0.93–5.27)	0.039 *
VEIN			
AD (AU)	152.12 (36.53–162.92)	158.42 (142.46–173.78)	0.140
BDF (AU)	4.14 (2.57–5.25)	4.12 (2.56–5.03)	0.949
MD (%)	5.95 (4.34–6.58)	5.50 (4.09–6.44)	0.322
MDRT (secs)	19.9 (17.0–22.7)	20.0 (18.0–22.3)	0.892
MC (%)	1.37 (0.38–1.72)	1.64 (0.39–1.99)	0.409
MCRT (secs)	21.4 (19.7–24.5)	21.5 (18.7–25.3)	0.907
DA (%)	7.32 (5.10–8.04)	7.09 (4.92–9.25)	0.685
bFR (%)	3.11 (1.26–4.74)	3.02 (1.41–4.19)	0.850

(↓ FMD—reduced ≤5% FMD group, Controls—FMD 7–10%). Values quoted in mean (IQR). * Significant values (*p* < 0.05). Abbreviations: AD: absolute diameter; BDF: baseline diameter fluctuation; MD: maximum dilation; MDRT: reaction time to reach maximum diameter to flicker stimulation; MC: maximum constriction; MCRT: reaction time to maximum constriction post flicker; DA: dilation amplitude; bFR: baseline-corrected flicker response.

## Data Availability

Data supporting this research available upon request from the corresponding author.
